# Cantilevers: Multi-Tool in Orthodontic Treatment

**DOI:** 10.3390/dj10070135

**Published:** 2022-07-19

**Authors:** Malgorzata Bilinska, Kasper Dahl Kristensen, Michel Dalstra

**Affiliations:** Section of Orthodontics, Department of Dentistry and Oral Health, Aarhus University, Vennelyst Boulevard 9, 8000 Aarhus, Denmark; kdki@dent.au.dk (K.D.K.); michel.dalstra@dent.au.dk (M.D.)

**Keywords:** orthodontics, cantilevers, statically determine system, segmented arch technique

## Abstract

This review aims to discuss and illustrate various uses of cantilevers to solve multiple clinical issues and prove their versatility. Cantilevers are commonly used in the segmented arch technique, and they can be designed to solve various clinical problems with highly predictable results. Its design and shape can modify the various combinations of vertical and horizontal forces. The novel trend is to combine cantilevers with skeletal anchorage. Cantilevers offer a very simple and statically determined force system. The advantage is the control over side effects, which normally occur on the anchor teeth and the occlusion. The disadvantages include possible side effects on the anchorage unit, when the anchorage is poorly controlled. The review highlights the clear benefits of cantilever use in complex corrections of single teeth, segments, and entire arch with a diminished effect on the dentition, also with the use of skeletal anchorage. With their simple and easily tailored design, these springs can be called an orthodontic multi-tool.

## 1. Introduction

**Figure 1:**
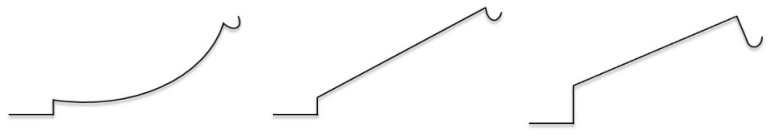
Different cantilever designs (activated). From the left: deep curve, tip back, and utility arch.

The application of well-defined biomechanical force systems allows to predict and control the tooth movement, according to the laws of equilibrium. The segmented arch technique was described by Charles Burstone in 1962 [1] and, in many cases, provides several clinical advantages over continuous archwires [2]. Understanding and the application of basic biomechanics aids to improve the orthodontic appliance efficiency, reduce possible side effects, and may shorten the overall treatment time. Cantilevers are commonly used in the segmented arch technique. Their design can solve various clinical problems with highly predictable results. However, combining cantilevers and straight-wire technique to move single displaced teeth decrease the risk of displacement of the well-positioned teeth [3]. As a rule, avoid connecting “the good with the bad”: severely displaced or impacted teeth with continuous archwire aids to prevent serious side effects. The system needs less frequent reactivations due to a low load/deflection rate and long span among the attachment points [4]. Cantilevers are most commonly made from titanium and molybdenum alloy (TMA) wire, but can also be made from stainless steel (SS). TMA can withstand more deflection than SS before permanent deformation occurs. It enables the creation of cantilevers with simpler designs and in most cases saves time during clinical procedures. TMA’s stiffness and modulus of elasticity provide a controlled force system and individualized tooth movement [5]. The cantilever’s design and shape can modify the various combinations of vertical and horizontal force. The use of a curved cantilever provides combined retraction and intrusion, while utility-shaped produces protrusive and intrusive forces [6,7] (Figure 1). In addition, the point of activation influences the force decay pattern over time [8].

**Figure 2:**
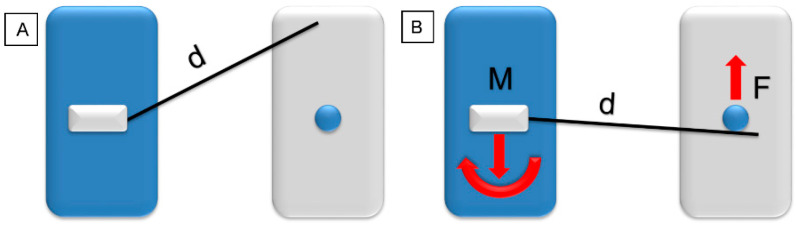
Model of a statically determined force system (blue: anchorage unit with cantilever; grey: point of application). (**A**) Cantilever in a neutral position, length (d). (**B**) Activated cantilever a single force (F), and a force and a moment (M) on the other side (red arrows).

A force system delivered by orthodontic appliances can be either statically determined or statically indetermined. In a statically determined force system, all forces and moments can be calculated using the force and moment equilibrium equations and are therefore predictable. For a statically indetermined force system, little is known from both a qualitative and quantitative point of view. A cantilever spring is a very simple and statically determined design. A single force is generated on the mesial end of the one-point contact. On the distal end, there is a reactive force in the opposite direction. These two forces generate a couple that has to be countered by a reactive moment for the sake of equilibrium (Figure 2).

A cantilever is a universal tool, but it is sometimes overlooked in clinical practice. This review aims to discuss and illustrate various uses of cantilevers to solve multiple clinical issues and prove their versatility.

## 2. Anchorage Considerations

**Figure 3:**
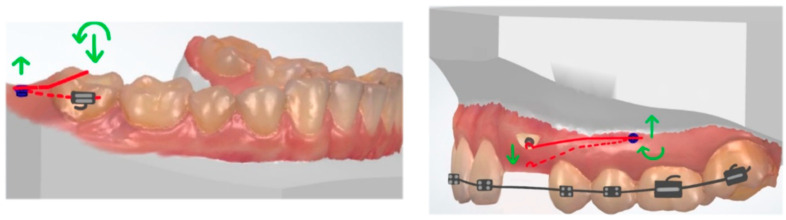
(**Left**): Placement of a TAD in line with or parallel to the dentition: no torquing moments on the TAD occurs. (**Right**) TAD is placed perpendicular to the long axis of the dentition; counterclockwise or clockwise torquing moments occurs (red: cantilever, green: force and moment).

The classic anchorage unit consists of a segment with an increasing number of teeth. The anchorage unit can also be reinforced with an intraoral appliance, for example, a transpalatal arch. The vertical extrusive force can be counteracted by the use of high-pull headgear, but occlusal forces could also partially prevent extrusion [9]. The novel trend is to combine cantilevers with skeletal anchorage (temporary anchorage devices, TADs, or miniscrews). In the literature, various applications of TAD-anchored one-couple systems are described. The advantage is the control over side effects, which normally occur on the anchor teeth and the occlusion. Using TADs does not reduce the need for biomechanical considerations, but reduces some of the obstacles. It is particularly advantageous when the correction of a single tooth is needed and the patient does not desire comprehensive orthodontic treatment. In the treatment of impacted teeth, the bonding of fully fixed appliance can be postponed until the impaction is resolved and possible ankylosis is ruled out [10]. When the cantilever is directly attached to the TADs, it can serve as a direct anchorage. To reduce wire play in the screw head, the cantilever’s size should match the dimensions of the slot [11]. As an indirect anchorage, skeletal anchorage stabilizes the anchorage unit. TADs can support the transpalatal arch (TPA) anchorage to prevent side effects on the molars [11]. The placement of a TAD in line with or parallel to the dentition minimizes torquing moments on the screw. When the TAD is placed perpendicular to the long axis of the dentition (buccal or palatal cortex), counterclockwise or clockwise torquing moments occur on the TAD and lead to its failure [12] (Figure 3).

## 3. Impacted Teeth

The management of ectopic teeth represents one of the greatest challenges in orthodontics. In the treatment of ectopic canines with a statically determined system, the cantilever’s force line of action can be adjusted according to the treatment need. For a buccally impacted canine, the choice would be either to extrude and mesialize/distalize the canine, while palatally impacted need extrusive and buccal pull-force components. In the case of bilateral impaction, traction can be provided simultaneously for both impacted teeth [13,14,15]. The use of light forces reduces the risk of complications and respects bone biology. The generated force of 25–30 cN extrudes a canine over a wide activation range [14,16]. A cantilever should be attached to the canine with a single-point contact to avoid the couple. The alternative is introducing the compensating toe-in bend when it is attached to the bracket slot [4]. The extrusion of the canine can be also achieved with a vertical tube-supported cantilever spring, as described by Vijayashree and Pai [17]. Supplementary anchorage with different appliance types or TADs decreases the stress levels on the adjacent teeth [18]. Both the biomechanics and force direction of the canine movement are necessary. Poorly controlled orthodontic extrusion may lead to the root resorption of adjacent teeth and introduced moments may cause unwanted rotations [19].

### 3.1. Buccal Impaction

**Figure 4:**
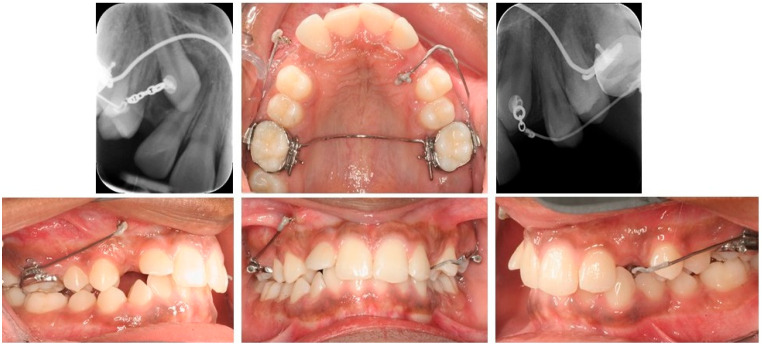
Bilateral canine impaction: (**right**) buccal and (**left**) palatal. Right maxillary canine: traction with 0.017 × 0.025″ TMA cantilever activated for extrusion. Left maxillary canine: cantilever is inserted into the auxiliary tube of an upper molar, activated for canine extrusion and buccal displacement.

A single extrusive force for the eruption of a buccally impacted canine can be easily generated with, e.g., a 0.017″ × 0.025″ TMA cantilever from the auxiliary tube of the first molar, attached to the canine with a single-point contact (Figure 4). 

**Figure 5:**
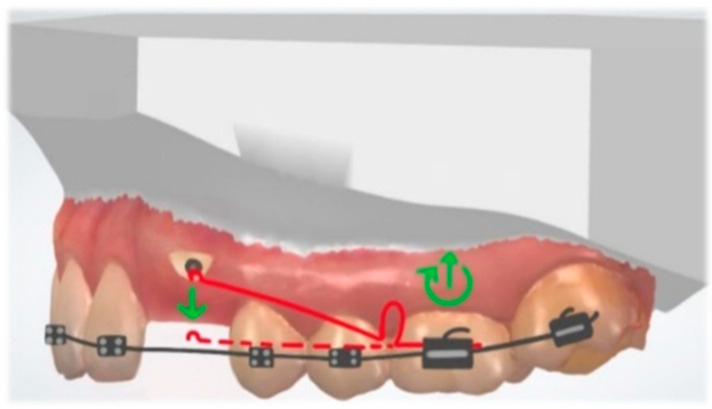
Cantilever with a loop activated for buccally impacted canine extrusion (red: cantilever, green: force and moment).

Katiyar et al. described the cantilever for buccally impacted canines with a closing loop, positioned mesially to the first molar. Its activation provides distal retraction of the canine, if needed [20] (Figure 5). After the canine is positioned in the arch, a box loop can be used to produce first- and second-order corrections while continuing the vertical eruption [21].

### 3.2. Palatal Impaction

**Figure 6:**
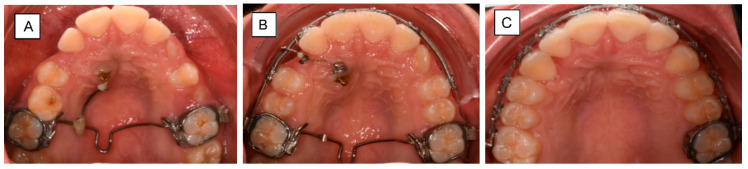
Traction of a palatally impacted right maxillary canine. (**A**) Canine extrusion with 0.017 × 0.025″ TMA cantilever inserted into the welded sheet on TPA, activated for extrusion. (**B**) Cantilever placed into the auxiliary tube of an upper molar, activated for canine extrusion and buccal displacement. (**C**) The canine is aligned into its final position.

The traction of the palatally impacted canines includes the tooth eruption out of the palate followed by buccal movement into the final arch position. A cantilever can be inserted into the molar buccal auxiliary tube and attached to the canine [14,22,23]. An alternative option, to avoid occlusal interference, is to attach the cantilever to the lingual sheet of the molar [4,14]. A cantilever could be also inserted into the welded sheet on the TPA (Figure 5). The described methods do not incorporate anchorage reinforcement. In prolonged canine traction, side effects (molar tipping and intrusion) will be observed if the anchorage unit is not well designed. To reduce side effects, a TPA can be employed (Figure 6) [24]. Nakandakari et al. described the canine traction with two cantilevers: one welded to the TPA, activated for extrusion, and a second one attached to the auxiliary molar tube, activated for buccal movement [24]. Tepedino et al. described the TPA with a soldered stainless-steel cantilever, similar to the helical torsion spring. The delivered force magnitude depends on the amount of activation, the wire diameter and the wire length. The length of the wire can be changed with the introduction of loops [25]. According to Tepedino et al., patients’ facial divergence and muscular activity have no impact on the force level for palatally impacted maxillary canines traction [16].

**Figure 7:**
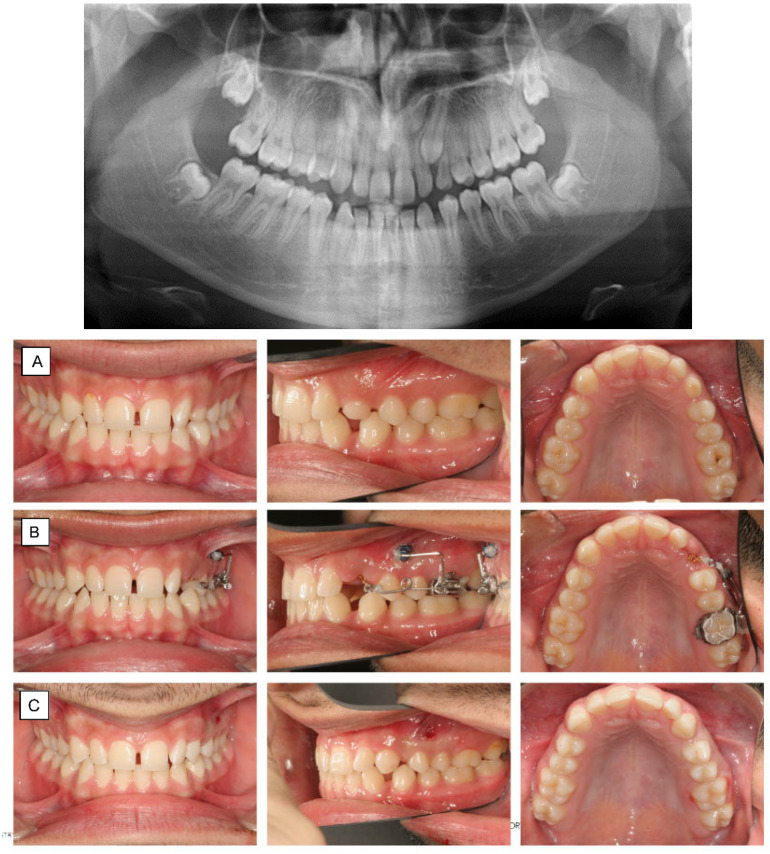
Panoramic x-ray shows ectopic tooth 23 in a 16-year-old patient with only slight resorption of 63 (**A**) Palatally impacted left maxillary canine. The patient was satisfied with the smile-esthetic with the diastemas. Therefore, a sectional appliance was chosen for correction of position of 23 without changing neither the occlusion or position of the rest of the teeth. (**B**) Canine extrusion with 0.017 × 0.025″ TMA cantilever inserted into the tube of the molar, activated for extrusion and buccal displacement. TAD is applied to stabilize indirectly the molar (anchorage unit). (**C**) The canine is aligned into its final position.

TADs can serve as direct and indirect anchorage. Thebault et al. and Heravi et al. described direct impacted canine traction with a cantilever attached to two TADs. The use of two miniscrews eliminates clockwise and counter-clockwise effects on the TADs during activation and deactivation and reduces the failure risk [10,11]. Annarumma et al. evaluated the traction of impacted canines attached to a double miniscrew and cantilever system only. Different cantilever designs were used to obtain canine extrusion and distalization, and to improve the torque. Skeletal anchorage allowed the tooth movement, without stressing the anchorage of the posterior teeth. The simplicity of the approach make the segmented method a good alternative in the treatment of canine impaction [26].The cantilever can be also attached both to the anchorage tooth unit and to the TAD, as a multi-attachment appliance. Insertion in the auxillary tube of a molar aids to reduce the moment acting on the head of the screw [11]. As an indirect anchorage, the TAD is used to stabilize the anchorage unit. The cantilever is attached to the auxiliary tube of the dental unit; the use of skeletal anchorage prevents side effects on the adjacent teeth (Figure 7).

When the palatally impacted canine is positioned in the arch, torque correction is needed in the finishing stage. Gandini et al. described the appliance to correct buccolingual inclination of teeth, which provide a large buccal root movement with minor crown dislocation. A 0.017 × 0.025 TMA cantilever is placed into a slot of the canine bracket bonded to the palatal or lingual surface of the tooth and attached to the transpalatal bar or the lingual arch as one point of contact. Depending on the initial position of the tooth, the buccolingual inclination can be corrected within 5 to 8 months [27].

## 4. Deep Bite Correction

**Figure 8:**
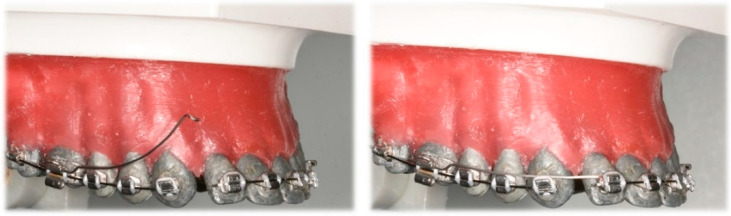
Three-piece intrusion arch. Deep curve shape cantilevers activated for anterior segment intrusion cantilevers: (**Left**) activated, before ligation; (**Right**) ligated distally to lateral incisors.

The three-piece intrusion base arch consists of anterior and posterior segments and two cantilevers, activated for intrusion (Figure 8).

It has a low force–deflection rate usually under 10 cN/mm due to the large distance between the auxiliary tube of the molar and the incisor brackets. When the force is applied at 90 degrees to the occlusal plane, the line of action can pass through the center of resistance of the incisors. It is achieved when the point of attachment is placed correctly and no flaring of the teeth occurs [28]. The use of light constant forces enables the intrusion of teeth with minimal side effects on the posterior anchor units. When intrusive forces increase, more root resorption occurs without changing tooth movement rate. Therefore, the force levels should be kept as low as possible. The desired magnitude depends on the number of teeth included in the intruded segment and their size. For the upper arch, about 60 cN of force for four incisors should be applied to the upper incisors [28]. Van Steenbergen et al. concluded that maxillary incisors could be intruded with forces of 10 to 20 cN per tooth. There was no difference whether 40 or 80 cN was used, for intrusion rate, extrusion of buccal segments, and change in intermolar width [9]. According to Burstone, the key to anchorage control is to maintain low-magnitude forces and use a rigid posterior segment, including a lingual arch or TPA [28]. The combination of intrusion and incisors retraction is clinically desirable when the overbite and overjet are increased as is often the case in perio-ortho patients. Melsen et al. evaluated the force system delivered by the SS and TMA cantilevers with an eccentrically placed helix loop. Three-piece intrusion mechanics allow the lateral displacement of the point of force application and the line of action passes closer to the center of resistance [29]. In the case of deep bite and flared incisors, Shroff et al. described a three-piece base arch, used together with Class I elastics, to correct deep bite and retract the incisors. Bilaterally placed tip back springs, fabricated from 0.017 × 0.025″ TMA wire, were placed to deliver intrusive force [30].

The mini-implant-supported three-piece Burstone base arch had a pronounced effect on the intrusion of flared four maxillary incisors with a clinically insignificant amount of root resorption. The technique was modified to integrate TADs to overcome the complications of the conventional anchorage protocol. The increased distal force helps to avoid unscrewing the TADs [31]. Mini plates could also serve as a cantilever attachment for anterior intrusion, as described by Thebault et al. [11].

## 5. Open Bite

**Figure 9:**
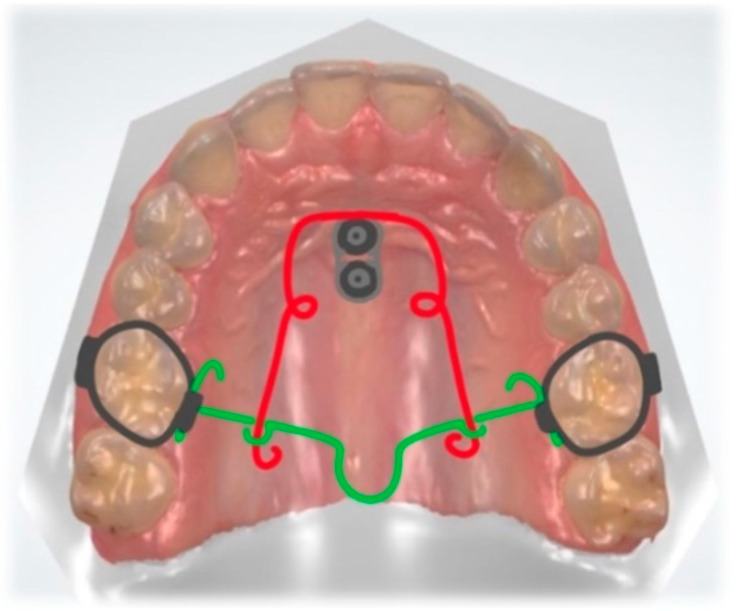
Mouse trap appliance. Lever arms connected to TADs, activated for molar intrusion (red). Transpalatal arch (green).

In some cases, an anterior open bite can be corrected with dental extrusion. However, stability in retention should always be considered. Kuhlberg described the system with two cantilevers connected to the anterior segment and anchorage unit, which works opposite the three-piece intrusion arch. A passive TPA was placed to prevent third-order movement of the molars [32]. Wilmes et al. [33] described the open bite correction with molar intrusion with the Mousetrap appliance (Figure 9). Lever arms connected to two mini-implants inserted in the anterior palate are activated for molar intrusion. To avoid tipping of the molars, TPA is placed. When the appliance is deactivated, the distal ends of the lever arms are located cranially to the molars’ centers of resistance (CR). The lever arms are activated with downward displacement and a constant intrusive force is delivered [33].

Flieger et al. presented a similar appliance to Mousetrap. The main difference is the placement of two Jet Screws, inserted half of the distance of the perpendicular line segment from the raphe to the palatal cusp tip of the first bicuspid. Posterior intrusion was achieved through distally extended cantilevers fabricated out of 16 × 22 stainless-steel wire, connecting the screw with maxillary molars [34]. Nojima et al. described open bite correction with the use of cantilevers and skeletal anchorage. TADs were placed in the middle of the palate and on the buccal alveolar bone, between the maxillary first molar and second premolar. Intrusive force was provided with 0.018 × 0.025″ TMA transpalatal arch with tear drop loops, tied to the bracket slot of the mini-implant and the palatal tubes of the maxillary molars. On the buccal side, intrusion was obtained with a 0.018 × 0.025″ TMA cantilever connected to the buccal TAD and molar auxiliary tube [35]. 

## 6. Intrusion

**Figure 10:**
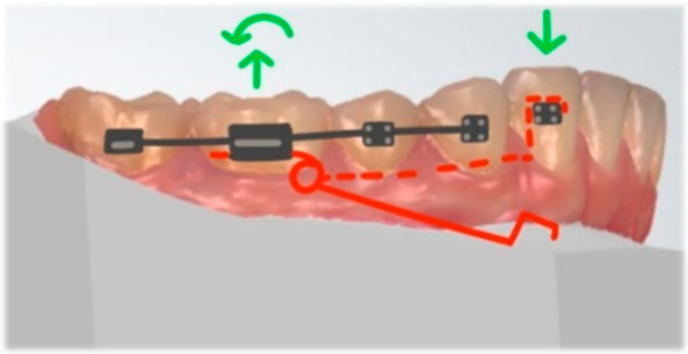
Cantilever activated for canine intrusion, placed on the top of the mandibular canine bracket (red: cantilever, green: force and moment).

The intrusion of a single tooth can be achieved with statically determined mechanics. According to the literature, 50 percent of the patients with a deep bite have overerupted mandibular canines [36]. In a finite element study by Caballero et al., the effects of a cantilever for intrusion were studied. Here, the cantilever was inserted into the auxiliary tube of the molar and placed on the top of the mandibular canine bracket (Figure 10). Since the force application point is localized on the labial side, a significant amount of labiolingual force occurs. However, the application of a 6-degree toe-in bend to prevent buccal and lingual crown tipping and produce pure intrusion of the canine was shown to be effective [3]. It is advised to attach the cantilever to the occlusal surface of a canine bracket. The insertion of a cantilever in a bracket slot produces an undesirable couple at the bracket slot [37]. Force application on the lingual side allows the achievement of almost pure intrusion while the bucco-lingual tooth inclination is maintained. Toe-in bend applied on a cantilever aid to achieve the pure intrusion of a mandibular canine. Its angle value depends on the height of the canine cusp [38]. A similar technique is used for the intrusion of the maxillary canine. When an auxiliary molar tube is not available, a cantilever can be inserted into the cross tube and ligated to the canine bracket [39]. Chandhoke et al. described a cantilever anchored on two buccal TADs for the correction of an overerupted second molar. The transpalatal arch controlled the transverse during the molar intrusion. The second molar was significantly intruded without buccal tipping [12].

## 7. Space Closure

**Figure 11:**
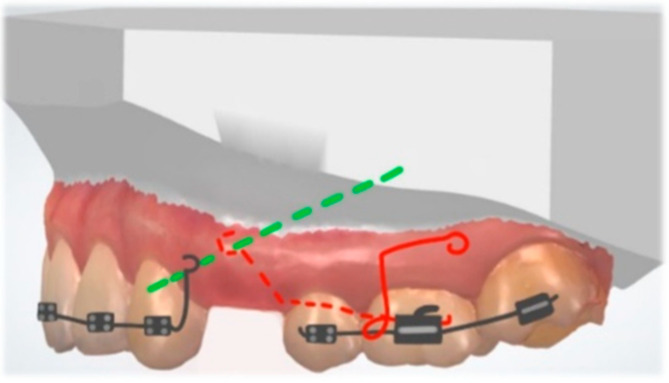
Statically determinate retraction system for space closure. Cantilever (red) is activated for anterior segment retraction (green: line of action).

Choy et al. [40] designed the statically determinate retraction system for space closure in extraction therapy. It consists of passive rigid stabilizing units and active retraction springs. The anterior and buccal stabilizing units are made of rigid stainless-steel wire, reinforced with a transpalatal arch. Distal extension with a hook is localized on the anterior stabilizing arch, about six mm superior to the canine bracket slot. A single-force cantilever arm made of 0.017 × 0.025″ TMA alloy wire is inserted into the molar tube for the retraction of the anterior segment (Figure 11). The cantilever spring and the anterior segment’s extension hook were connected with a ligature. A low load-deflection rate of the cantilever spring provides a constant force: at full activation, the spring delivered 163 cN with a load-deflection rate of 6 cN/mm [40].

## 8. Occlusal Cant

It is important to set a diagnosis between an incisal cant and an occlusal cant. Incisal can be corrected with well-controlled, determinate force systems, while the correction of the occlusal cant is more challenging to treat [41]. In growing patients, the treatment approach may be the controlled eruption of buccal segments; in adults, often only surgical correction is feasible. Deluke et al. proposed the treatment of lower incisal cant with a 0.017″ × 0.025″ TMA cantilever, attached from the first molar auxiliary tube to the main archwire between the central and lateral incisors. Sectioning the main wire allowed the unilateral intrusion of the front segment [41]. Musilli et al. proposed a cantilever, similar in shape to a Ricketts utility arch with a modification of distal hooks attached to the continuous archwire bilaterally between the second premolar and first molar. This canting spring produces intrusion on one side of the anterior segment and extrusion on the other. Since it is applied on the continuous arch as an overlay system, the correction can be achieved without creating steps between the canines and the lateral incisors [42]. Chandhoke et al. described the use of skeletal anchorage and a cantilever for the correction of a mandibular cant with the simultaneous closure of the lateral open bite and transverse correction. In a one-couple system, the force skewed the mandibular arch in the axial plane, reducing the canine overjet and correcting the dental midline [12]. According to van Steenbergen and Nanda, in the posterior occlusal plane cant, the posterior segment may be uprighted with the cantilever hooked to the anterior segment. Expected side effects are the extrusion of the buccal segment and unilateral intrusion of the anterior segment. The large tip-back moment on the buccal segment flattens the occlusal plane [43].

## 9. Asymmetry, Midline Correction

The correction of the midline discrepancies is important both for aesthetics and to achieve functional occlusion [44]. According to Nanda et al., the use of a cantilever is ideal in apical base discrepancies, when the aim is to upright tipped incisors and change their axial inclinations [45]. When the midline discrepancy is caused by tipping of the lower incisors, simple force applied at the crowns of the teeth will upright the incisors. In case when the bodily movement of incisors is needed, the cantilever shall be attached to the passive loop, extended apically to approximate the center of resistance of the incisor teeth [32]. Fiorelli et al. proposed the simultaneous treatment of deep bite and midline correction. The system consisted of two cantilevers and an anterior segment. One cantilever was activated for intrusive force delivered to an anterior segment, laterally to the maxillary lateral incisor. The tipping of the segment was counteracted by the horizontal force provided by the cantilever on the contralateral side [44]. Mittal et al. designed similar system for midline correction. Anterior segment with a vertical extension approximating the center of resistance is displaced with a 0.017″ × 0.025″ TMA cantilever, bent buccally and tied to the loop with an elastomeric chain. When activated, the system produces the force that results in an efficient midline correction through the pure translation of the anterior segment [46].

**Figure 12:**
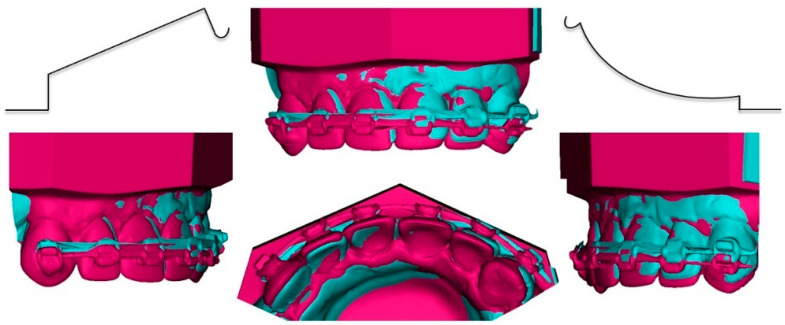
Asymmetric cantilever activation: activation of utility arch (right side of typodont) and deep curve cantilever (left side of typodont), resulting intrusion and displacement of anterior segment (before: pink; after: blue).

The experimental study by Bilinska and Dalstra revealed that different shapes of cantilevers produce vertical, but also horizontal forces. The cantilever with a deep curve shape produces retraction and lateral force, and the utility shape protraction and medial force. When the use of different shapes is attached to the sides of the front segment, the transversal force may facilitate the midline correction [7] (Figure 12).

## 10. Molar Uprighting

**Figure 13:**
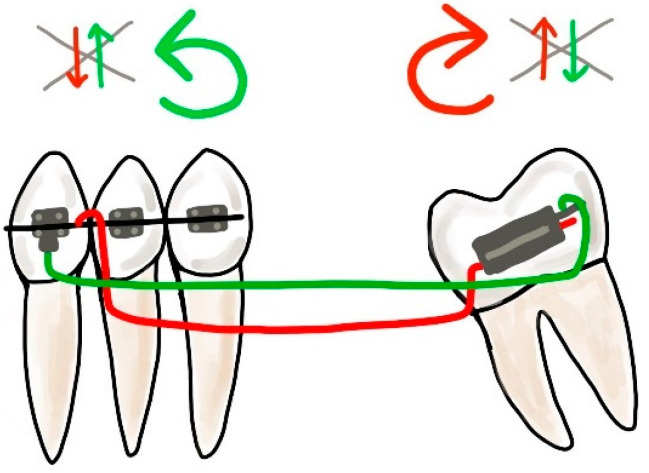
Double cantilever mechanics. The red cantilever, from the molar to the anterior teeth, generates extrusion, clockwise rotation on the molar, and intrusion on the anterior teeth (red arrows). In order to control the vertical forces, a second cantilever, from a tube in the anterior, is required. This green cantilever is placed distally to the molar and it produces molar intrusion and a counter-clockwise movement with extrusion on the anterior teeth (green arrows). The resultant forces cancel each other, and two opposite moments occur.

A classic biomechanical treatment modality for molar uprighting is the segmented approach. The cantilever inserted into the molar tube is hooked on the anterior teeth segment and generates extrusion and clockwise rotation on the molar, intrusion on the anterior teeth. To control the vertical forces, a double cantilever system may be used (Figure 13). As a result, we only have two opposite moments, on the molar and the anterior teeth segment [47].

Uprighting of the mesially tipped molars often differentiates between success and failure in periodontal and restorative treatments [48]. Khouw et al. described the helical uprighting spring. It is inserted into the molar tube and attached to the continuous wire, between the canine and premolar. The resultant force is extrusion; a moment with a distal direction aids to upright the tooth and lingual crown tipping. The effect on the anchorage unit should be taken into consideration: the intrusion and lingual tip of the premolars [48]. According to Kojima et al., introducing the bend in the cantilever towards the lingual direction reduces the stress on the anchor teeth, which possibly may reduce the side effects [49]. Ma et al. described the uprighting of the impacted second and third molar. In the first phase, the impacted third molar was distalized with a three-loop spring. The second molar was uprighted with the cantilever inserted into the impacted molar buccal tube and its free end was hooked onto the main archwire to produce an uprighting force. New bone apposition was observed after orthodontic extrusion distally to the adjacent tooth. The technique could be effective in the separation of third molars from the nerve proximity to provide safe extraction with the risk of neurosensory deficit [50,51]. Alessandri Bonetti et al. presented a disimpaction technique, called “orthodontic extraction” of the third molar with a cantilever, which would facilitate its extraction. Cantilever activated for extrusion is connected to the molar and anchorage unit. When the third molar extrudes, the distance between the roots and the mandibular canal increases. Its favorable position for the surgery reduces the risk of nerve damage [52].

“Kissing molars” describe a type of tooth impaction with two mandibular molars severely tipped and impacted with their occlusal surfaces positioned crown-to-crown, while the roots are pointing in opposite directions. The common treatment protocol is the extraction of impacted teeth. The treatment approach to upright and preserve the molars was described by Barros et al. The protocol included the use of torqued cantilever mechanics, where the torque arm moves the roots in a mesiodistal direction. The created mesiodistal moment of force is applied on the molar roots of the second and produces an uprighting effect [53]. A long cantilever arm can deliver a relatively low load-deflection rate and produces a force system to facilitate root uprighting [54]. In the described case, a second molar uprighting occurred mainly due to the torque movement, associated mostly with root repositioning [53].

Morita et al. discussed two different uprighting mechanics separately applied to the mesially tipped mandibular first and second molars. For the uprighting of the impacted and severally tipped first molar, the distal end of the cantilever was twisted to generate the sufficient uprighting moment. On the mesial end, the cantilever was directly attached to the TAD to counteract the extrusive force. The second molar was uprighted with a compression force with two step bends incorporated into a nickel–titanium archwire. The molar was tipped distally [55]. Chandhoke described the uprighting spring, which was anchored by TAD for the correction of mesially tipped lower right first and second molars. The cantilever was stabilized at the TAD, to avoid undesirable moments at the screw and its failure [12]. Methods on molar uprighting with the use of TADs were described by Musilli et al. [47]. The molar can be uprighted with a cantilever attached to the TAD in the retromolar area. The force system clinically produces a moment and an intrusive force on the molar. In the described approach, no other additional appliances are required. When the molar is uprighted with a long cantilever attached to the anterior segment, a TAD can be placed mesially and ligated to the molar to provide a vertical force control. This system has the advantages of the classic double cantilever approach and is more comfortable for the patient [47]. 

## 11. Dental Transposition

Mechanics for correction of the dental transposition should be planned individually, reducing the potential risks and side effects. In the transposition of canines and the first premolar, the canine is usually displaced in the mesiobuccal direction between the first and second premolars. The first premolar is often tipped distally and displaced in a mesiopalatal direction [56]. A segmented treatment approach was presented by Laino et al., when tooth impaction and dental transposition were corrected with the use of different cantilever configurations [2]. Capelozza Filho et al. presented a case report of the clinical approach to unilateral tooth transposition of a maxillary canine and first premolar. The first premolar was displaced in the distal and palatal direction with a 0.019 × 0.025″ TMA cantilever. Following the premolar correction, the maxillary canine was mesialized into its final position with torque control [57]. Lorente et al. corrected incomplete maxillary canine–first premolar transposition with a cantilever spring coupled to the auxiliary band tube. The anchorage unit was reinforced with a TPA. The canine was pulled in a mesial and apical direction. The aim was to bring the canine to the widest part of the dentoalveolar process to minimize the amount of periodontal recession [58]. In the case of transposition of the canine and lateral incisor, the canine was displaced buccally with the cantilever, while the lateral incisor was mesialized into the right place in the arch. The use of a cantilever with loops provided the controlled movement of transposed teeth and control of the anchorage unit [59]. Fu et al. used an innovative cantilever and simplified mechanics to correct an ectopic central incisor and the transposed canine–lateral incisor without periodontal complications. The cantilever was attached to a TAD and pulled the impacted tooth canine buccally and distally toward its normal position. The use of skeletal anchorage provided sufficient anchorage [60].

## 12. Single Tooth Extrusion

**Figure 14:**
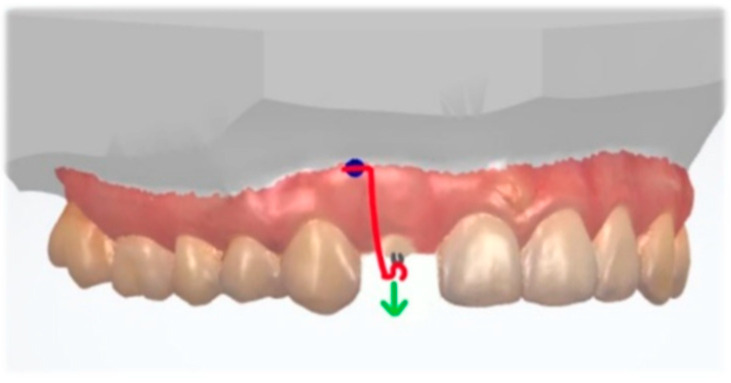
Extrusive force (green) applied along the long tooth axis with a cantilever (red) connected to the TAD (blue).

Skeletal anchorage with a cantilever can be also used for a forced eruption for teeth with subgingival defects, such as root fractures and subgingival cervical caries. Noh and Park described a system with a TAD and a cantilever, attached to the root of lateral incisor. An extrusive force was applied along the long tooth axis. In this method, there is no need to bond brackets to other teeth to correct the target tooth [61] (Figure 14).

Kumar et al. described the successful management and prosthetic rehabilitation of a complicated horizontal root fracture in the mandibular left first premolar and mandibular lateral incisor. When the coronal fragment is extremely mobile, endodontic treatment, decoronation, and orthodontic extrusion provide an easy approach for functional and aesthetic rehabilitation. After root canal treatment, the root of the incisor was extruded with a 0.017 × 0.025″ TMA cantilever attached to the molar and the root post. The extrusion was followed with the crown restoration. The first premolar was extruded with a helical coiled 0.014 inch NiTi (nickel–titanium) wire attached to canine and molar. The helix was tied to the hooked end of the post with ligature wire. Both teeth were stabilized for 8 weeks prior to prosthetic rehabilitation [62].

## 13. Molar Distalization

**Figure 15:**
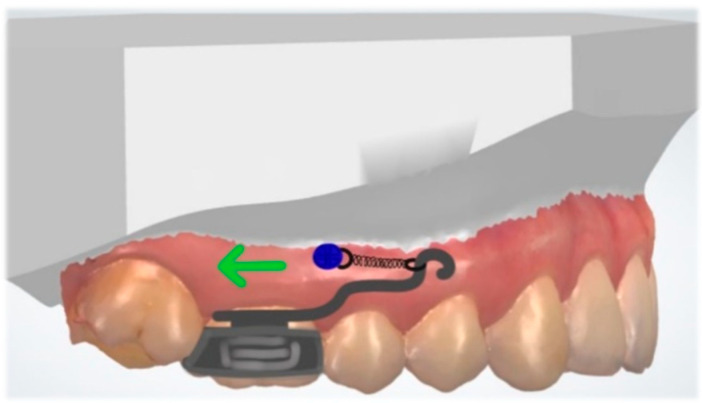
Unilateral molar distalization with cantilever connected to the TAD with a closed-coil spring (green: force).

Molar distalization is commonly used for the non-extraction treatment of unilateral or bilateral Class II malocclusions. Direct skeletal anchorage helps to avoid anchorage loss and unwanted side effects on the dentition. Vilanova et al. described a miniscrew-anchored cantilever. TAD is placed on the buccal side, between the roots of the second premolar and upper first molar. A 0.014″ SS cantilever is inserted into the molar tube. TAD and the cantilever are connected with a nickel–titanium closed-coil spring with 200 g of force (Figure 15). The appliance deliver a horizontal force as close as possible to the CR of the upper first molar and result in a distal bodily movement [63].

## 14. Discussion

This review article discussed several applications of one-couple systems, both the classic approach and using skeletal anchorage. We highlighted the clear benefits of cantilevers used in complex corrections of single teeth, segments, and entire arch with a diminished effect on the dentition. The use of skeletal anchorage provides benefits in terms of anchorage reinforcement and reduces the complexity of orthodontic treatment. Combining TADs with cantilevers increase treatment possibilities.

Modern technologies could aid to improve cantilever design and improve the treatment flow. In the last decade, the interest in the field of robotic wire bending and robotic customization of CAD/CAM appliances has increased [64]. To improve the precision, cantilevers could be designed in the software and bend indirectly by the robots. It could reduce the chairside time and improve precision. Liu et al. presented a collaboration between the robot and external bending machine. The method combines task and motion planning for a robot to curve metal wires into 3D shapes. The described system can bend different 3D shapes with satisfying performance [65].

In the modern treatment approach, 3D printed appliances are becoming more and more popular, with regards for printed orthodontic appliances [66]. For the future treatment modalities, cantilevers could be designed in the software and printed, as it is possible to print beta titanium alloys for biomedical applications [67]. Polymer 3D printing is a developing technology offering printing low-cost functional parts with diverse capabilities and properties [68]. Orthodontic appliances, such as 3D printed distalizers and various auxiliaries (e.g., power-arms), can be produced with additive manufacturing with biocompatible photopolymers [69]. To introduce this technique, the polymer cantilever should have similar mechanical properties to TMA alloys. According to Guerrero-Gironés et al., the assessment of the biocompatibility of 3D printing and conventional resins revealed no major differences [70]. Possibility to produce the tooth-shade or transparent cantilevers would increase aesthetics. In the field of modern technologies in regard to cantilevers production, there is still a room for further investigation. Nowadays, chairside bending offers fast and cost-effective treatment approach.

## 15. Conclusions

With the correct force system and biomechanical understanding, cantilevers generate a predictable force system to solve the variability of orthodontic problems. With their simple and easily tailored design, these springs can be called an orthodontic multi-tool.

## Data Availability

Not applicable.

## References

[1] Burstone C.J. (1962). Rationale of the segmented arch. Am. J. Orthod..

[2] Laino A., Cacciafesta V., Martina R. (2001). Treatment of tooth impaction and transposition with a segmented-arch technique. J. Clin. Orthod..

[3] Caballero G.M., Carvalho Filho O.A., Hargreaves B.O., Brito H.H., Magalhaes Junior P.A., Oliveira D.D. (2015). Mandibular canine intrusion with the segmented arch technique: A finite element method study. Am. J. Orthod. Dentofac. Orthop..

[4] Fischer T.J., Ziegler F., Lundberg C. (2000). Cantilever mechanics for treatment of impacted canines. J. Clin. Orthod..

[5] Gurgel J.A., Pinzan-Vercelino C.R., Powers J.M. (2011). Mechanical properties of beta-titanium wires. Angle Orthod..

[6] Dalstra M., Melsen B. (1999). Force systems developed by six different cantilever configurations. Clin. Orthod. Res..

[7] Bilinska M., Dalstra M. (2022). The Effect of Symmetric and Asymmetric Loading of Frontal Segment with Two Curved Cantilevers: An In Vitro Study. Dent. J..

[8] Jacob H.B., Gonzaga A.S., Trinh B., Le E.T., English J.D. (2021). Effects of stress relaxation in beta-titanium cantilevers used in orthodontic mechanics. Dent. Press J. Orthod..

[9] van Steenbergen E., Burstone C.J., Prahl-Andersen B., Aartman I.H. (2005). The influence of force magnitude on intrusion of the maxillary segment. Angle Orthod..

[10] Heravi F., Shafaee H., Forouzanfar A., Zarch S.H., Merati M. (2016). The effect of canine disimpaction performed with temporary anchorage devices (TADs) before comprehensive orthodontic treatment to avoid root resorption of adjacent teeth. Dent. Press J. Orthod..

[11] Thebault B., Dutertre E. (2015). Disimpaction of maxillary canines using temporary bone anchorage and cantilever springs. Int. Orthod..

[12] Chandhoke T.K., Nanda R., Uribe F.A. (2015). Clinical applications of predictable force systems, part 2: Miniscrew anchorage. J. Clin. Orthod..

[13] Sukh R., Singh G.P., Tandon P. (2014). Interdisciplinary approach for the management of bilaterally impacted maxillary canines. Contemp. Clin. Dent..

[14] Yadav S., Upadhyay M., Uribe F., Nanda R. (2013). Mechanics for treatment of impacted and ectopically erupted maxillary canines. J. Clin. Orthod..

[15] Potrubacz M.I., Chimenti C., Marchione L., Tepedino M. (2018). Retrospective evaluation of treatment time and efficiency of a predictable cantilever system for orthodontic extrusion of impacted maxillary canines. Am. J. Orthod. Dentofac. Orthop..

[16] Tepedino M., Iancu-Potrubacz M., Grippaudo C., Chimenti C., Lagana G. (2018). Does muscular activity related to vertical facial divergence influence the time needed for orthodontic extrusion of palatally impacted maxillary canines? A retrospective study. J. Clin. Exp. Dent..

[17] Vijayashree U.H., Pai V. (2017). Canine extrusion with a vertical tube supported cantilever spring. Apos Trends Orthod..

[18] Zeno K.G., El-Mohtar S.J., Mustapha S., Ghafari J.G. (2019). Finite element analysis of stresses on adjacent teeth during the traction of palatally impacted canines. Angle Orthod..

[19] Fleming P.S., Sharma P.K., DiBiase A.T. (2010). How to...mechanically erupt a palatal canine. J. Orthod..

[20] Katiyar R., Singh G.P., Tandon P. (2012). A cantilever spring for alignment of buccally impacted canines. J. Clin. Orthod..

[21] Patel S., Cacciafesta V., Bosch C. (1999). Alignment of impacted canines with cantilevers and box loops. J. Clin. Orthod..

[22] Paduano S., Spagnuolo G., Franzese G., Pellegrino G., Valletta R., Cioffi I. (2013). Use of cantilever mechanics for impacted teeth: Case series. Open Dent. J..

[23] Paduano S., Cioffi I., Iodice G., d’Anto V., Riccitiello F., Pellegrino G., Valletta R. (2014). Correction of multiple canine impactions by mixed straightwire and cantilever mechanics: A case report. Case Rep. Dent..

[24] Nakandakari C., Goncalves J.R., Cassano D.S., Raveli T.B., Bianchi J., Raveli D.B. (2016). Orthodontic Traction of Impacted Canine Using Cantilever. Case Rep. Dent..

[25] Tepedino M., Chimenti C., Masedu F., Potrubacz M.I. (2018). Predictable method to deliver physiologic force for extrusion of palatally impacted maxillary canines. Am. J. Orthod. Dentofac. Orthop..

[26] Annarumma F., D’Emidio M., Rodi G., Battista G., Papi G., Migliorati M. (2021). The effectiveness of miniscrews in the three-dimensional control of a palatal impacted canine: “Canine Only” approach. Case report. Int. Orthod..

[27] Gandini L.G., Gandini M.R., Amaral R.M. (2010). Continuous torque system with control of the reaction unit. Am. J. Orthod. Dentofac. Orthop..

[28] Burstone C. (2001). Biomechanics of Deep Overbite Correction. Semin. Orthod..

[29] Melsen B., Konstantellos V., Lagoudakis M., Planert J. (1997). Combined intrusion and retraction generated by cantilevers with helical coils. J. Orofac. Orthop..

[30] Shroff B., Lindauer S.J., Burstone C.J., Leiss J.B. (1995). Segmented approach to simultaneous intrusion and space closure: Biomechanics of the three-piece base arch appliance. Am. J. Orthod. Dentofac. Orthop..

[31] Albelasy N.F., Montasser M.A., Hafez A.M., Abdelnaby Y.L. (2022). Effects on root axes and resorption of simultaneous intrusion and retraction of maxillary central and lateral incisors using mini-implant supported three-piece burstone base arch: A prospective observational study. Int. Orthod..

[32] Kuhlberg A. (2001). Cantilever Springs: Force System and Clinical Applications. Semin. Orthod..

[33] Wilmes B., Vasudavan S., Stocker B., Willmann J.H., Drescher D. (2015). Closure of an open bite using the ‘Mousetrap’ appliance: A 3-year follow-up. Aust. Orthod. J..

[34] Flieger S., Ziebura T., Kleinheinz J., Wiechmann D. (2012). A simplified approach to true molar intrusion. Head Face Med..

[35] Nojima L.I., Barreto B.C.T., Vargas E.O.A., Starling C.R., Nojima M.D.C.G. (2022). A clinical approach to managing open-bite malocclusion associated with severe crowding. Am. J. Orthod. Dentofac. Orthop..

[36] El-Dawlatly M.M., Fayed M.M., Mostafa Y.A. (2012). Deep overbite malocclusion: Analysis of the underlying components. Am. J. Orthod. Dentofac. Orthop..

[37] Burstone C.R. (1977). Deep overbite correction by intrusion. Am. J. Orthod..

[38] Thote A.M., Sharma K., Uddanwadiker R.V., Shrivastava S. (2017). Optimum pure intrusion of a mandibular canine with the segmented arch in lingual orthodontics. Biomed. Mater. Eng..

[39] Vu B.T., Soroushian S. (2013). Single-tooth intrusion with a cross tube and a cantilever spring. J. Clin. Orthod..

[40] Choy K., Pae E.K., Kim K.H., Park Y.C., Burstone C.J. (2002). Controlled space closure with a statically determinate retraction system. Angle Orthod..

[41] Deluke M., Uribe F., Nanda R. (2006). Correction of a canted lower incisal plane. J. Clin. Orthod..

[42] Musilli M., Grampone F., Melsen B. (2014). A new auxiliary spring for correction of a canted incisal plane. J. Clin. Orthod..

[43] van Steenbergen E., Nanda R. (1995). Biomechanics of orthodontic correction of dental asymmetries. Am. J. Orthod. Dentofac. Orthop..

[44] Fiorelli G., Melsen B., Modica C. (2001). Differentiated orthodontic mechanics for dental midline correction. J. Clin. Orthod..

[45] Nanda R., Margolis M.J. (1996). Treatment strategies for midline discrepancies. Semin. Orthod..

[46] Mittal T., Singh H., Kapoor P., Sharma P. (2020). Dental midline correction using a cantilever spring: A novel approach. Int. J. Orthod. Rehabil..

[47] Musilli M., Marsico M., Romanucci A., Grampone F. (2010). Molar uprighting with mini screws: Comparison among different systems and relative biomechanical analysis. Prog. Orthod..

[48] Khouw F.E., Norton L.A. (1972). The mechanism of fixed molar uprighting appliances. J. Prosthet. Dent..

[49] Kojima Y., Mizuno T., Fukui H. (2007). A numerical simulation of tooth movement produced by molar uprighting spring. Am. J. Orthod. Dentofac. Orthop..

[50] Ma Z., Yang C., Zhang S., Xie Q., Shen Y., Shen P. (2014). Orthodontic extrusion of horizontally impacted mandibular molars. Int. J. Clin. Exp. Med..

[51] Tay A.B., Go W.S. (2004). Effect of exposed inferior alveolar neurovascular bundle during surgical removal of impacted lower third molars. J. Oral. Maxillofac. Surg..

[52] Bonetti G.A., Bendandi M., Laino L., Checchi V., Checchi L. (2007). Orthodontic extraction: Riskless extraction of impacted lower third molars close to the mandibular canal. J. Oral. Maxillofac. Surg..

[53] Barros S.E., Janson G., Chiqueto K., Ferreira E., Rosing C. (2018). Expanding torque possibilities: A skeletally anchored torqued cantilever for uprighting “kissing molars”. Am. J. Orthod. Dentofac. Orthop..

[54] Sawicka M., Racka-Pilszak B., Rosnowska-Mazurkiewicz A. (2007). Uprighting partially impacted permanent second molars. Angle Orthod..

[55] Morita Y., Koga Y., Nguyen T.A., Yoshida N. (2020). Biomechanical considerations for uprighting impacted mandibular molars. Korean J. Orthod..

[56] Allen W.A. (1967). Bilateral transposition of teeth in two brothers. Br. Dent. J..

[57] Filho L.C., Mde A.C., An T.L., Bertoz F.A. (2007). Maxillary canine—First premolar transposition. Angle Orthod..

[58] Lorente C., Lorente P., Perez-Vela M., Esquinas C., Lorente T. (2020). Orthodontic management of a complete and an incomplete maxillary canine-first premolar transposition. Angle Orthod..

[59] Gebert T.J., Palma V.C., Borges A.H., Volpato L.E. (2014). Dental transposition of canine and lateral incisor and impacted central incisor treatment: A case report. Dent. Press J. Orthod..

[60] Fu P.S., Wang J.C., Wu Y.M., Huang T.K., Chen W.C., Tseng Y.C., Tseng C.H., Hung C.C. (2013). Unilaterally impacted maxillary central incisor and canine with ipsilateral transposed canine-lateral incisor. Angle Orthod..

[61] Noh H.K., Park H.S. (2019). An efficient and noncompliant method for forced eruption with microimplants that is bracket free, and its long-term stability. J. Am. Dent. Assoc..

[62] Kumar G., Verma N., Parashar S. (2019). Management of Subgingival Root Fracture with Decoronation and Orthodontic Extrusion in Mandibular Dentition: A Report of Two Cases. Contemp. Clin. Dent..

[63] Vilanova L., Henriques J.F.C., Patel M.P., Da Costa Grec R.H., Aliaga-Del Castillo A. (2020). The Miniscrew-Anchored Cantilever: A Simple Molar Distalizer. J. Clin. Orthod..

[64] Adel S., Zaher A., El Harouni N., Venugopal A., Premjani P., Vaid N. (2021). Robotic Applications in Orthodontics: Changing the Face of Contemporary Clinical Care. Biomed. Res. Int..

[65] Liu R., Wan W., Isomura E.T., Harada K. (2022). Metal wire manipulation planning for 3D curving—How a low payload robot can use a bending machine to bend stiff metal wire. arXiv.

[66] van der Meer W.J., Vissink A., Ren Y. (2016). Full 3-dimensional digital workflow for multicomponent dental appliances: A proof of concept. J. Am. Dent. Assoc..

[67] Pellizzari M., Jam A., Tschon M., Fini M., Lora C., Benedetti M. (2020). A 3D-Printed Ultra-Low Young’s Modulus β-Ti Alloy for Biomedical Applications. Materials.

[68] Arefin A.M.E., Khatri N.R., Kulkarni N., Egan P.F. (2021). Polymer 3D Printing Review: Materials, Process, and Design Strategies for Medical Applications. Polymers.

[69] Thurzo A., Urbanová W., Novák B., Waczulíková I., Varga I. (2022). Utilization of a 3D Printed Orthodontic Distalizer for Tooth-Borne Hybrid Treatment in Class II Unilateral Malocclusions. Materials.

[70] Guerrero-Gironés J., López-García S., Pecci-Lloret M.R., Pecci-Lloret M.P., Lozano F.J.R., García-Bernal D. (2022). In vitro biocompatibility testing of 3D printing and conventional resins for occlusal devices: Biocompatibility of 3D printing and conventional resins. J. Dent..

